# Efficacy of Intranasal Dexmedetomidine Premedication as an Adjunct on Intubation Process in Paediatric Patients: A Randomized, Double-blind, Placebo-controlled Trial

**DOI:** 10.4274/TJAR.2026.252306

**Published:** 2026-06-26

**Authors:** Octava Prima Arta, Raihanita Zahra, Christopher Kapuangan, Indro Mulyono, Arif H. M. Marsaban, Aldy Heriwardito

**Affiliations:** 1 Indonesia Universty Faculty of Medicine Cipto Mangunkusumo Hospital, Department of Anaesthesiology and Intensive Care, Jakarta, Indonesia

**Keywords:** Dexmedetomidine, intranasal administration, paediatric intubation, premedication, sedation quality, opioid sparing

## Abstract

**Objective:**

To evaluate the effect of a single intranasal dose of dexmedetomidine (2 µg kg^-1^) on intubation efficacy, mask acceptance, sedation quality, intubation duration, and intraoperative fentanyl consumption in children undergoing elective surgery.

**Methods:**

A randomized, double-blind, placebo-controlled trial was conducted in 40 American Society of Anaesthesiologists I-II children aged 1-6 years who were scheduled for elective surgery. Participants were randomized to receive either dexmedetomidine (n = 20) or 0.9% sodium chloride placebo (n = 20). All patients received intravenous midazolam as a routine premedication. Primary outcomes were mean arterial pressure (MAP) and heart rate (HR), measured at baseline and post-intubation; these were compared to assess intubation efficacy. Secondary outcomes included mask acceptance, sedation quality, intubation time, and total fentanyl consumption. Data were analyzed using the independent-samples t-test or the Mann-Whitney U test (*P* < 0.05).

**Results:**

Dexmedetomidine maintained MAP (1.34**±**19.59%) versus a significant rise with placebo (21.95**±**26.36%; *P*=0.008) and limited HR increase (1.6% vs. 22.2%; *P* < 0.001). Mask acceptance and adequate sedation were significantly higher (*P* < 0.001). Intubation duration did not differ (median 39 seconds vs. 44 seconds; *P*=0.267). Fentanyl consumption was significantly lower (1.10 vs. 2.15 µg kg^-1^; *P* < 0.001).

**Conclusion:**

A single intranasal dose of dexmedetomidine provides superior intubation efficacy, improves mask acceptance and sedation quality, and yields a significant opioid-sparing effect.

**Main Points**

• A single preoperative intranasal dose of dexmedetomidine (2 µg kg^-1^) effectively attenuated the hypertensive and tachycardic response to tracheal intubation, maintaining significantly more stable mean arterial pressure and heart rate compared with placebo.

• The dexmedetomidine group demonstrated significantly better mask acceptance and sedation quality prior to anaesthesia induction, facilitating a smoother, more cooperative induction.

• The use of intranasal dexmedetomidine resulted in nearly a 50% reduction in intraoperative fentanyl consumption (median 1.10 µg kg^-1^ vs. 2.15 µg kg^-1^), highlighting its potent analgesic and adjunctive properties.

## Introduction

Children undergoing tracheal intubation are prone to respiratory and systemic complications because of heightened airway reactivity to mechanical or chemical stimuli, painful laryngoscopy, positive-pressure ventilation, and aspiration. These events are a major source of perioperative morbidity and can precipitate cardiac arrest.^1, 2^ In addition, intubation-related systemic adverse events range from mild (bronchial or esophageal intubation, dysrhythmias, hypertension, epistaxis) to severe (tooth trauma, pneumothorax, laryngospasm, profound hypotension, aspiration, or cardiac arrest with or without return of spontaneous circulation).^3^

Perioperative anxiety and distress are also highly prevalent in the paediatric population, with up to 75% of children experiencing significant preoperative anxiety that may translate into adverse behavioral and emotional outcomes postoperatively.^4^ Effective anxiolysis and sedation before induction can attenuate airway reflexes, improve mask acceptance, and shorten induction time, thereby enhancing intubation conditions and reducing complications.^5, 6^ Traditional premedication routes—oral and intravenous—are often impractical in children because of poor cooperation, unpredictable absorption, and the discomfort associated with IV cannulation.^7^

The intranasal route is a non‑invasive drug administration pathway that produces local, systemic, and central nervous system effects. Intranasal drug delivery offers several advantages, including ease of administration, rapid onset of action, and minimal pain perception.^7, 8^ Intranasal dexmedetomidine, a highly selective α_2_‑adrenergic agonist, offers a non‑invasive alternative with rapid onset, minimal discomfort, and a favorable safety profile that includes sedation, anxiolysis, and mild analgesia without respiratory depression. It is associated with minimal complications, and nasal irritation is the most commonly reported adverse effect.^8, 9^

Recent meta‑analyses comparing intranasal dexmedetomidine with intranasal midazolam in paediatric patients have demonstrated superior satisfaction with parental separation, higher mask acceptance rates, and reduced postoperative pain and nasal irritation.^10^ Over the past decade, studies have consistently shown that intranasal dexmedetomidine (1-2 µg kg^-1^) can diminish stress responses during intubation in children.^11^ Although previous studies have established the sedative benefits of intranasal dexmedetomidine in children, its direct effects on paediatric intubation have not been investigated.

This study aimed to evaluate the efficacy of adjunct intranasal dexmedetomidine premedication in paediatric intubation, focusing on intubation efficacy, mask acceptance, sedation depth, intubation duration, and opioid consumption-outcomes that directly influence patient safety and procedural efficiency. To our knowledge, this study is the first to evaluate its impact on intubation conditions, providing novel evidence for a potentially safer, non-invasive premedication strategy. We hypothesized that, compared with placebo, intranasal dexmedetomidine would improve intubation efficacy, enhance mask acceptance and sedation quality, stabilize hemodynamic responses, and reduce opioid requirements.

## Methods

This randomized, double-blind, placebo-controlled clinical trial evaluated the adjunctive effect of intranasal dexmedetomidine premedication on intubation efficacy and anaesthesia outcomes in paediatric patients. This study was approved by the Ethics Committee of the Indonesia Universty Faculty of Medicine, Dr. Cipto Mangunkusumo National General Hospital (approval no: KET-1568/UN2.F1/ETIK/PPM.00.02/2024, date: 28.10.2024). The study adhered to the Declaration of Helsinki and relevant national regulations and was registered at ClinicalTrials.gov (NCT06991647, date: 18.03.2025). Written informed consent was obtained from the parents or legal guardians of all enrolled children. This manuscript follows the CONSORT reporting guidelines.

The trial was conducted from November to December 2024 in the operating rooms of Cipto Mangunkusumo Hospital, Jakarta, Indonesia. Eligible participants were children aged 1-6 years, with American Society of Anaesthesiologists (ASA) physical status I-II, who were scheduled for elective surgery under general anaesthesia. Exclusion criteria included an anticipated difficult airway, active oral or nasal infection, neurodevelopmental disorders (e.g., cerebral palsy, attention-deficit/hyperactivity disorder), and an inability to obtain intravenous access.

Participants were allocated to the intervention or control arm using simple randomization. Allocation sequences were generated online and placed in opaque, sealed envelopes (SNOSE technique). An independent research assistant prepared the study medication, ensuring blinding of participants, clinicians, and outcome assessors.

The study used a Intranasal Mucosal Atomization Device (MAD) Nasal™ (MAD300, Teleflex Inc., Wayne, PA, USA) coupled with 1 mL syringes to deliver intranasal medication. The investigational drug was dexmedetomidine hydrochloride 100 µg mL^-1^, which was diluted with 0.9% sodium chloride (NaCl) to a total volume of 1 mL for each dose; the placebo solution consisted of 0.9% NaCl alone, administered in an identical volume. Standard anaesthesia equipment included a sevoflurane vaporizer, fentanyl, and atracurium for induction and muscle relaxation, along with non-invasive blood pressure monitoring, electrocardiogram, pulse oximetry, and capnography, all integrated into a calibrated anaesthesia workstation. Mask acceptance and sedation quality were assessed throughout the peri-intubation period using the paediatric anaesthesia behaviour (PAB) score and the COMFORT Behaviour Scale, respectively.

Thirty minutes before induction, the intervention group received intranasal dexmedetomidine 2 µg kg^-1^ (diluted to 0.5 mL per nostril), while the control group received an equal volume of intranasal 0.9% NaCl as placebo. All children in both groups received intravenous midazolam 0.05 mg kg^-1^ as routine premedication, immediately after intranasal dexmedetomidine or placebo administration. Baseline mean arterial pressure (MAP) and heart rate (HR) were recorded before drug administration. Upon arrival in the operating theatre, PAB and COMFORT scores were documented. Anaesthesia induction proceeded with sevoflurane 2% inhalation, followed by titrated fentanyl (initial 1 µg kg^-1^, with additional 1 µg kg^-1^ boluses if MAP or HR increased >20% during intraoperative monitoring). Inhalation induction was preferred because it had minimal effects on MAP and HR compared with IV iSnduction. Atracurium 0.5 mg kg^-1^ was used for muscle relaxation. Direct laryngoscopy was performed, and endotracheal tube size was calculated using the formula: diameter (mm) = 4+ (age in years /4). Intubation duration was measured from laryngoscope insertion to detection of end-tidal carbon dioxide. MAP, HR, and total fentanyl dose required for intubation were recorded immediately after tube placement. Intubation efficacy was defined as the ability to attenuate hemodynamic responses, measured as low variability in MAP and HR across groups. Episodes of bradycardia were treated according to paediatric advanced life support guidelines (atropine 0.02 mg kg^-1^, maximum 0.5 mg). All adverse events related to intranasal dexmedetomidine administration, anaesthetic induction, or intubation were systematically recorded throughout the perioperative period.

A priori power analysis (α=0.05, power=80%) based on an expected difference in MAP of 10 mmHg and pooled standard deviation (SD) of 7.5 mmHg (from Naushad et al.^12^) indicated a minimum of 36 participants (18 per group). Although the minimum sample size was also estimated using multiple studies assessing various outcomes, this calculation yielded the largest sample size requirement. To compensate for a potential 10% dropout, 40 participants were enrolled consecutively until the target sample size was reached.

The primary outcome was premedication efficacy, measured by MAP and HR at baseline and post-intubation. Secondary outcomes included mask acceptance, sedation quality, intubation duration, and total fentanyl consumption.

### Statistical Analysis

Data were entered into a blinded case report form and analyzed using the Statistical Package for the Social Sciences version 20 (IBM, Armonk, NY, USA). Continuous variables were expressed as mean ± SD or median (interquartile range) based on distribution (Kolmogorov-Smirnov test). Between-group comparisons used the independent-samples t test or Mann-Whitney U test, and within-group comparisons used the paired-samples t test or Wilcoxon signed-rank test, as appropriate; categorical variables were analyzed using the χ^2^ test or Fisher’s exact test. A two-tailed *P* < 0.05 was considered statistically significant.

## Results

**Figure 1:**
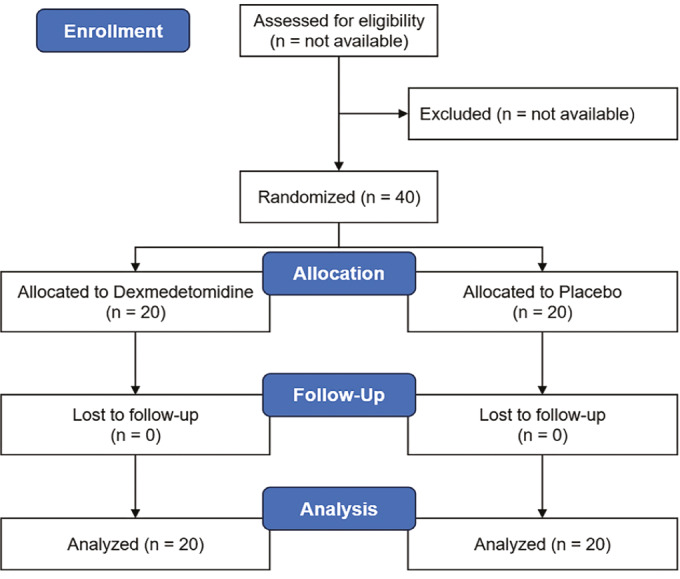
CONSORT flow diagram.

A total of 40 paediatric patients who met the inclusion criteria and consented to participate were enrolled in the study. The participants were randomly assigned to two groups using a double-blind design: a placebo group and an intranasal dexmedetomidine group, with comparable median ages (2 years for dexmedetomidine vs. 3 years for placebo) and similar gender distribution (Table 1). The majority of patients were ASA II, indicating that they were generally healthy, and fasting durations were similar, with just over half fasting <12 hours. Nutritional status was largely comparable, although the dexmedetomidine group included a higher proportion of underweight children and contained the sole overweight participant, whereas the placebo arm had more normal-weight participants. The distribution of surgical specialties was well-balanced across groups, covering general, paediatric, urologic, plastic, orthopedic, ear, nose and throat, and dental procedures, with comparable numbers in each category. No adverse events related to the intranasal route of administration or to dexmedetomidine side effects were reported during the sampling period. Additionally, no participants dropped out during the course of the study (Figure 1).

Table 2 shows that the baseline MAP was similar between the dexmedetomididine (72.35±15.95 mmHg) and placebo (69.30±17.04 mmHg) groups, indicating comparable initial hemodynamic conditions prior to intervention. Following intubation, the placebo group experienced a marked increase in MAP to 82.60±19.46 mmHg, which was significantly greater than the dexmedetomidine group (71.70±14.00 mmHg). The percentage change from baseline further highlighted this difference, with the placebo group showing a 21.95±26.36% rise compared with only 1.34±19.59% in the dexmedetomidine group (*P*=0.008). Within-group analyses confirmed a statistically significant elevation in MAP for the placebo group (*P*=0.002), whereas the dexmedetomidine group showed a non-significant change in MAP (*P*=0.852).

Regarding HR, the dexmedetomidine group demonstrated a higher baseline HR of 121.35±20.68 beats per minute (bpm) than the placebo group (105.65±14.72 bpm), which may be attributed to the younger average age of patients in the dexmedetomidine arm. After intubation, the placebo group exhibited a notable increase in HR to 132.00±12.07 bpm, significantly surpassing the post-intubation HR of the dexmedetomidine group (123.80±16.01 bpm). The percentage change in HR from baseline differed significantly between groups; the placebo group showed a median increase of 22.22%, versus a minimal increase of 1.57% in the dexmedetomidine group (*P* < 0.001). Furthermore, within-group comparisons revealed a highly significant elevation in HR in the placebo group (*P* < 0.001), whereas HR changes in the dexmedetomidine group were not statistically significant (*P*=0.268).

Table 3 shows significantly greater mask acceptance in the dexmedetomidine group than in the placebo group, particularly in the highest acceptance category labeled “Happy.” All Patients in the dexmedetomidine group were rated “Happy” (100%), whereas none of the patients in the placebo group were classified at this level. This corresponds to an odds ratio of 168.20 [95% confidence interval (CI) =7.4-3,818.5; *P* < 0.001], indicating that dexmedetomidine was associated with a markedly higher likelihood of achieving the highest mask acceptance. Conversely, the “Sad” and “Mad” categories, indicating poorer mask acceptance, were predominantly observed in the placebo group.

Sedation quality was also markedly better in the dexmedetomidine group, with significantly greater sedation scores reflecting more effective anxiolysis and sedation-an effect up to 51-fold greater than placebo (95% CI=7.6-343.7; *P* < 0.001). Despite differences in sedation and cooperation, intubation duration did not differ significantly between groups (median time =39 second in the dexmedetomidine group vs. 44 second in the placebo group; *P*=0.267). Finally, fentanyl consumption was significantly lower in the dexmedetomidine group (*P* < 0.001), with a median of 1.10 µg kg^-1^, than in the placebo group (median=2.15 µg kg^-1^).

## Discussion

Dexmedetomidine preserves baseline MAP by activating central α2-adrenergic receptors, suppressing norepinephrine release, reducing sympathetic outflow, and enhancing parasympathetic tone. This autonomic modulation and its analgesic effects blunt stress responses during intubation and prevent hypertensive surges in children, who are particularly vulnerable to blood pressure fluctuations.^13, 14, 15^ Naushad et al.^12^ similarly reported that paediatric patients receiving dexmedetomidine maintained more stable MAP levels than those premedicated with opioids, emphasizing the importance of hemodynamic stability in reducing the risk of end-organ injury during invasive procedures.^12^

Our findings are consistent with the above findings, supporting the utility of dexmedetomidine not only for sedation but also for protection of paediatric patients from harmful blood pressure fluctuations during intubation and other invasive procedures. These advantages position dexmedetomidine as a safer and more effective premedication option in paediatric clinical practice.^12, 15^

Dexmedetomidine maintains HR control by activating central α_2_-adrenergic receptors, which reduces central sympathetic outflow and augments vagal activity, thereby directly lowering HR.^16^ Its analgesic effects indirectly support HR control by minimizing pain-induced sympathetic activation. By blunting autonomic stress responses and reducing adrenergic stimulation through hemodynamic and analgesic mechanisms, dexmedetomidine contributes to the induction of effective bradycardia and the modulation of pain.^13, 17^

This study’s findings regarding the level of mask acceptance are consistent with those of Zhang et al.,^9^ who reported that intranasal dexmedetomidine resulted in higher rates of mask acceptance than midazolam (81.47% vs. 60.92%; *P* < 0.01). Dexmedetomidine’s sedative effects promote greater cooperation in paediatric patients during anaesthetic mask application and reduce resistance to anaesthesia induction.^9^ Our results corroborate these findings, further supporting the use of dexmedetomidine as a premedication that effectively reduces preoperative anxiety in paediatric patients.^10^

A meta-analysis conducted by Lang et al.,^18^ comparing dexmedetomidine and midazolam across 34 randomized controlled trials involving 2,281 paediatric patients, found that dexmedetomidine was associated with better sedation outcomes during parent-child separation, with a risk ratio (RR) of 0.78 [95% CI (0.65-0.92)]. Improved sedation quality with dexmedetomidine contributes to reduced preoperative anxiety, a frequent challenge in paediatric anaesthesia. Sedation assessments in these studies used structured tools, such as six-point sedation scales to evaluate aspects including patient cooperation and mask acceptance.

This study’s findings align with prior evidence showing that dexmedetomidine provides high-quality sedation without serious adverse effects, such as respiratory depression or postoperative agitation-side effects that are more commonly associated with midazolam. Furthermore, the same meta-analysis found that dexmedetomidine was associated with a significantly lower incidence of postoperative agitation [RR 0.31; 95% CI (0.24-0.41)], underscoring its dual benefit as both an effective sedative and a modulator of postoperative behavior.^18^

Although intranasal dexmedetomidine may facilitate a more favorable sedative state, similar intubation times between the two groups suggest that the duration of intubation is influenced by factors beyond premedication. Variables such as the use of muscle relaxants, operator experience, intubation technique, and the procedural environment also play substantial roles in determining the time required for intubation.

A study by Castle et al.^19^ supports this interpretation, in which the use of various sedative premedications and intubation aids did not significantly affect the duration of intubation. Instead, operator experience was identified as a more critical determinant of procedural duration. Their study also emphasized that the primary benefit of premedication lies in improving patient comfort rather than reducing procedural time.^19^ Nevertheless, that study highlighted that improving patient comfort during intubation can reduce resistance and procedural difficulty, enhance clinician satisfaction, and potentially lower the risk of complications. Although intubation duration may not be a direct indicator of dexmedetomidine’s efficacy, its ability to foster a calm, cooperative patient environment remains a compelling rationale for its use in paediatric anaesthesia protocols. Further studies may be warranted to investigate the combined impact of pharmacologic premedication and operator training on clinical outcomes.^19^

Hu et al.^20^ identified an optimal intranasal dose of 2 µg kg^-1^ dexmedetomidine in paediatric patients, demonstrating significant analgesic efficacy and a marked reduction in opioid requirements. Similarly, Kumar et al.^21^ found that intranasal dexmedetomidine at doses of 1-2 µg kg^-1^ provided prolonged analgesic effects due to stable plasma concentrations achieved through slow absorption across the nasal mucosa. Both studies support the role of intranasal dexmedetomidine as an effective opioid-sparing analgesic strategy.

In our study, the dexmedetomidine group required significantly lower doses of fentanyl than the control group. This reduction reflects dexmedetomidine’s ability to provide additional analgesia, thereby decreasing reliance on opioids such as fentanyl during intubation. This analgesic effect not only reduces pain perception but also suppresses excessive nociceptive responses, thereby making dexmedetomidine a valuable analgesic adjuvant in paediatric anaesthetic procedures.^16^

The baseline characteristics of the two groups were highly similar in terms of age, gender, ASA status, fasting duration, and surgical distribution, suggesting effective randomization and a low risk of confounding from baseline imbalances. This comparability strengthens the validity of any observed differences in outcomes between the groups. The absence of adverse events associated with intranasal dexmedetomidine and the lack of study dropouts indicate a favorable safety and tolerability profile in this paediatric cohort, supporting its potential use in preoperative settings. These findings align with existing literature on the safety of intranasal dexmedetomidine and underscore the feasibility of its administration in young children undergoing elective surgery.

### Study Limitations

This study has several limitations. First, intubation was not performed by the same operator for all participants, potentially introducing variability in mechanical manipulation and nociceptive stimulation. The study population was limited to paediatric patients with ASA I-II status, which may limit the generalizability of the findings to children with higher ASA classifications or severe comorbidities. Other variables—such as operator skill, procedural environment, and anaesthetic protocols beyond premedication—were not analyzed. Dexmedetomidine was administered as a fixed weight-based dose, despite possible interindividual pharmacologic variability. The observation period was restricted to the intubation procedure, without assessment of postoperative outcomes or adverse events. Conducting the study at a single center and using 0.9% NaCl as the sole placebo, without comparison with other common premedications such as ketamine or propofol, further limits the generalizability and comparative interpretation of the findings.

## Conclusion

Adjunct intranasal dexmedetomidine premedication (2 µg kg^-1^) markedly provides superior efficacy in intubation, enhances preintubation sedation quality and mask acceptance, and produces a significant opioid-sparing effect in children undergoing airway instrumentation, while not prolonging intubation duration when compared with placebo. These findings support its use as a safe, non-invasive premedication to facilitate smoother induction of paediatric anaesthesia and to reduce intraoperative fentanyl requirements.

## Ethics

**Ethics Committee Approval:** This study was approved by the Ethics Committee of the Indonesia Universty Faculty of Medicine, Dr. Cipto Mangunkusumo National General Hospital (approval no.: KET-1568/UN2.F1/ETIK/PPM.00.02/2024, date: 28.10.2024).

**Informed Consent:** Written informed consent was obtained from the parents or legal guardians of all enrolled children.

## Figures and Tables

**Table 1. Patient Characteristics:** 

**Variable**	**Treatment arm**		***P* value**
	**Dexmedetomidine** **(n = 20)**	**Placebo** **(n = 20)**	
Age (years)	2.00 (1.00, 4.00)	3.00 (1.00, 5.00)	0.524^a^
Gender	-	-	0.465^b^
Male	14 (46.7)	16 (53.3)	-
Female	6 (60.0)	4 (40.0)	-
Asa classification	-	-	0.723^b^
I	6 (54.5)	5 (45.5)	-
II	14 (48.3)	15 (51.7)	-
Nutritional status	-	-	0.219^b^
Underweight	5 (62.5)	3 (37.5)	-
Normal	13 (43.3)	17 (56.7)	-
Overweight	2 (100.0)	0 (0)	-
Fasting duration	-	-	0.548^b^
<12 hours	19 (51.4)	18 (48.6)	-
≥12 hours	1 (33.3)	2 (66.7)	-
Surgical specialty	-	-	0.761^b^
General paediatric	6 (50.0)	6 (50.0)	-
Urologic	8 (53.3)	7 (46.7)	-
Plastic	3 (60.0)	2 (40.0)	-
Orthopedic	2 (66.7)	1 (33.3)	-
Ear, nose, and throat	1 (25.0)	3 (75.0)	-
Dental	0 (0)	1 (100.0)	-

Values are median (1Q, 3Q) or n (%), ^a^: Mann-Whitney U test, ^b^: Pearson chi-square

ASA, American Society of Anesthesiologists

**Table 2. Premedication Efficacy Between Treatment Arms:** 

**Variable**	**Treatment arm**		***P* value** **(between groups)**
	**Dexmedetomidine** **(n = 20)**	**Placebo** **(n = 20)**	
Mean arterial pressure (mmHg)	-	-	-
Baseline	72.35±15.95	69.30±17.04	0.562^a^
Post-intubation	71.70±14.00	82.60±19.46	0.049^a^*
Percentage of change	1.34±19.59	21.95±26.36	0.008^a^*
P value (within groups)	0.852^c^	0.002^c^*	-
Heart rate (beats per minute)	-	-	-
Baseline	121.35±20.68	105.65±14.72	0.009^a^*
Post-intubation	123.80±16.01	132.00±12.07	0.075^a^
Percentage of change	1.57 (-4.18, 12.063)	22.22 (14.34, 30.66)	<0.001^b^*
P value (within groups)	0.268^d^	<0.001^d^*	-

Values are mean ± standard deviation or median (1Q, 3Q), ^a^: Independent-samples t test, ^b^: Mann-Whitney U test, ^c^: Paired-samples t test, ^d^: Wilcoxon signed-rank test, *: Statistically significant

**Table 3. Secondary Outcomes:** 

**Variable**	**Treatment arms**		***P* value and** **odds ratio**
	**Dexmedetomidine** **(n = 20)**	**Placebo** **(n = 20)**	
Mask acceptance	-	-	<0.001^a*^
Happy	14 (100.0)	0 (0)	168.20 (7.4-3818.5)
Sad	4 (40.0)	6 (60.0)	4.67 (0.7-32.7)
Mad	2 (12.5)	14 (87.5)	1.00
Sedation quality	-	-	<0.001^a*^
Adequate	18 (85.7)	3 (14.3)	51.0 (7.6-343.7)
Inadequate	2 (10.5)	17 (89.5)	1.00
Intubation duration (second)	39 (34, 59)	44 (39, 62)	0.267^b^
Fentanyl consumption (µg kg^-1^)	1.10 (1.10, 1.48)	2.15 (1.83, 2.58)	<0.001^b*^

Values are n (%) or median (1Q, 3Q), odds ratios for categorical data are presented as odds ratio (95% confidence interval), ^a^: Pearson chi-square, ^b^: Mann-Whitney U test, ^*^: Statistically significant
